# Supplementary figures and images for: Training understanding of reversible sentences: a study comparing language-impaired children with age-matched and grammar-matched controls
Source: PeerJ. 2014 Nov 4;2:e656. doi: 10.7717/peerj.656 (PMC4226637; doi:10.7717/peerj.656)

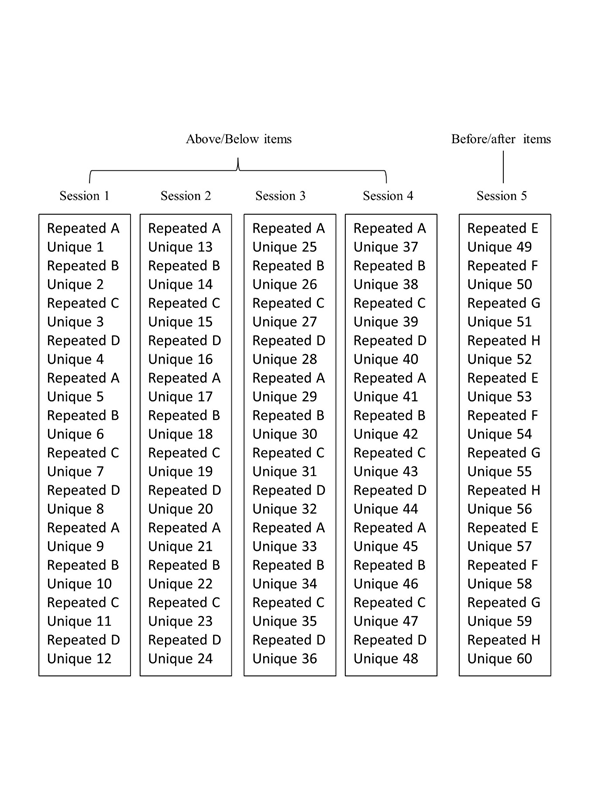

Supplement: Appendix S1 — Items marked Unique contain new nouns, whereas those marked Repeated have exactly the same words on each presentation. The four training sessions use the same preposition pair, whereas at post-test, the other preposition pair is presented. Sample sentences for Session 1: Repeated A: “The apple is above the horse”; Repeated B: “The chair is above the rabbit”; Repeated C: “The cow is below the soap”; Repeated D: “The ball is below the turtle”; Unique 1: “The baby is below the owl”; Unique 2: “The hedgehog is below the crown”; Unique 3: “The gate is below the butterfly”; Unique 4: “The orange is above the table”; Unique 5: “The book is above the lamp”; Unique 6: “The flag is below the bear”; Unique 7: “The zebra is below the cake”; Unique 8: “The bus is below the eye”; Unique 9: “The bike is above the fox”; Unique 10: “The bell is above the kite”; Unique 11: “The cushion is above the wall”; Unique 12: “The cup is below the door”. [file peerj-02-656-s001.png]
